# Biomarkers to Evaluate Androgen Deprivation Therapy for Prostate Cancer and Risk of Alzheimer’s Disease and Neurodegeneration: Old Drugs, New Concerns

**DOI:** 10.3389/fonc.2021.734881

**Published:** 2021-12-15

**Authors:** Vérane Achard, Kelly Ceyzériat, Benjamin B. Tournier, Giovanni B. Frisoni, Valentina Garibotto, Thomas Zilli

**Affiliations:** ^1^ Division of Radiation Oncology, Department of Oncology, Geneva University Hospitals and Faculty of Medicine, Geneva University, Geneva, Switzerland; ^2^ Division of Nuclear Medicine and Molecular Imaging, Diagnostic Department, Geneva University Hospitals, and NimtLab, Faculty of Medicine, Geneva University, Geneva, Switzerland; ^3^ Division of Adult Psychiatry, Department of Psychiatry, Geneva University Hospitals and Faculty of Medicine, Geneva University, Geneva, Switzerland; ^4^ Memory Clinic, Department of Rehabilitation and Geriatrics, Geneva University and University Hospitals, Geneva, Switzerland

**Keywords:** androgen deprivation therapy, prostate cancer, Alzheimer’s disease, dementia, functional brain imaging, biomarkers, blood

## Abstract

Androgen deprivation therapy (ADT) is a standard treatment for prostate cancer patients, routinely used in the palliative or in the curative setting in association with radiotherapy. Among the systemic long-term side effects of ADT, growing data suggest a potentially increased risk of dementia/Alzheimer’s disease in prostate cancer patients treated with hormonal manipulation. While pre-clinical data suggest that androgen ablation may have neurotoxic effects due to Aβ accumulation and increased tau phosphorylation in small animal brains, clinical studies have measured the impact of ADT on long-term cognitive function, with conflicting results, and studies on biological changes after ADT are still lacking. The aim of this review is to report on the current evidence on the association between the ADT use and the risk of cognitive impairment in prostate cancer patients. We will focus on the contribution of Alzheimer’s disease biomarkers, namely through imaging, to investigate potential ADT-induced brain modifications. The evidence from these preliminary studies shows brain changes in gray matter volume, cortical activation and metabolism associated with ADT, however with a large variability in biomarker selection, ADT duration and cognitive outcome. Importantly, no study investigated yet biomarkers of Alzheimer’s disease pathology, namely amyloid and tau. These preliminary data emphasize the need for larger targeted investigations.

## Introduction

Prostate cancer (PCa) is the most common cancer diagnosed among men in countries worldwide and it is the second leading cause of men cancer-related death (1). Androgen deprivation therapy (ADT) combined with chemotherapy or second-line generation androgen receptor inhibitors constitutes the mainstay treatment for patients with metastatic hormone-sensitive or castration resistant PCa and is frequently combined with curative radiotherapy as primary treatment for patients with localized/locally advanced disease or as salvage in the post prostatectomy setting, due to improvement with level-1 evidence in strong clinical outcomes such as overall survival (2–5). With the broadening of indications for ADT use, it is now estimated that up to 50% of patients newly diagnosed with PCa will receive ADT during the course of their disease management (6). Androgen ablation has a wide range of adverse effects, including hot flushes, sexual dysfunction, cardiovascular disease, metabolic syndrome, osteoporosis, and cognitive changes (7). The risk of neurocognitive disorders such as dementia and/or Alzheimer’s disease (AD) in patients with PCa who have received ADT has been recently studied through retrospective analyses of large databases with conflicting results (8–14). The mechanisms of the possible association between ADT and dementia or AD are still poorly understood. Aim of the present narrative review is to shed light on clinical and pre-clinical evidence supporting the potential link between androgen ablation and neurocognitive disorders, to explore the current AD biomarkers and to help clinicians integrate this risk for PCa patients care. We will first summarize the clinical results of the main studies assessing the risk of AD/dementia in PCa patients treated with ADT. Some of these studies (as well as a well-conducted meta-analysis of these studies) establish a correlation between ADT and dementia, but not all of them. However, these contradictory results can be balanced with the fact that imaging studies in PCa patient’s cohorts show changes in neural activation and brain structure when treated with ADT, and that preclinical data on small animals support an association between testosterone deprivation and AD pathogenesis. Secondly we will review the actual biomarkers of AD and see if they have been applied in this specific setting. Finally, we will discuss the clinical repercussions of this specific potential risk for PCa patients as well as the future perspectives in this field.

## Androgen Deprivation Therapy/Testosterone Depletion And Alzheimer’s Disease

### Use of ADT and Risk of Neurodegeneration and/or Alzheimer’s Disease in PCa Patients

Using clinical data from large electronic medical records, several cohort studies have analyzed the association between ADT and occurrence of cognitive impairment and AD. We will focus on the most recent and largest cohort studies results on the subject (**Table 1**). We will begin by the studies establishing a correlation between cognitive impairment/AD and ADT (9, 10, 12–14), then describe two studies which question this potential link (8, 11) and finally, we will present a recent meta-analysis of these observational cohort studies conducted by Sari Motlagh et al. (15).

**Table 1:** Retrospective cohort studies investigating association between androgen deprivation therapy and occurrence of cognitive impairment or Alzheimer’s disease in prostate cancer patients.

	Reference	PCa patientsn	Database	ADTn (%)	Non ADTn (%)	Type of ADT	Evaluated outcome	HRs of cognitive decline/dementia
**Positive studies**	**Hong 2020** (9)	17,425	Longitudinal Health Insurance Database for Catastrophic Illness Patients	12,740 (73.1%)	4,685 (26.9%)	bilateral orchiectomy or medical treatment (LHRH agonist, anti-androgens, or combination therapy)	Cognitive decline	HR = 1.54, 95% CI 1.29–1.84
	**Nguyen 2018** (13)	201,797	Surveillance, Epidemiology and End Results (SEER)-Medicare linked database of the National Cancer institute	94,528 (46.8%)	107,269 (53.2%)	LHRH agonists or antagonists	AD/Dementia	HR 1.16, 95% CI 1.13-1.20
	**Krasnova 2020** (12)	100,414	Surveillance, Epidemiology and End Results (SEER)-Medicare linked database of the National Cancer institute	37,911 (37.8%)	62,503 (62.2%)	bilateral orchiectomy or medical treatment	All cause Dementia AD	HR 1.17, 95% CI 1.07-1.27 HR 1.23, 95% CI 1.11-1.37
	**Jayadevappa 2019** (10)	154,089	Surveillance, Epidemiology and End Results (SEER)-Medicare linked database of the National Cancer institute	62,330 (40.5%)	91, 759 (59.5%)	bilateral orchiectomy or medical treatment	Dementia AD	HR = 1.20, 95% CI 1.17-1.24 HR = 1.14, 95% CI 1.10-1.18
	**Tae 2019** (14)	37,549	National Health Insurance Service database	24,929 (66.4%)	12,620 (33.6%)	bilateral orchiectomy or medical treatment (LHRH agonists, oral antiandrogens, oral estrogens)	Cognitive decline	HR, 1.169, 95% CI 1.077-1.270
**Negative studies**	**Baik 2017** (8)	1,238,879	fee–for-service Medicare beneficiaries	440,129 (35%)	798,750 (65%)	bilateral orchiectomy or medical treatment	AD Dementia	HR, 0.98, 95% CI 0.97-0.99 HR, 1.01, 95% CI 1.01-1.02
	**Khosrow-Khavar 2017** (11)	30,903	United Kingdom’s Clinical Practice Research Datalink	17,994 (58.2%)	12,909 (41.8%)	bilateral orchiectomy or medical treatment (LHRH agonists, oral antiandrogens, oral estrogens)	Dementia	HR 1.02, 95% CI 0.87-1.19

PCa, Prostate cancer; ADT, androgen deprivation therapy; HR, hazard ratio; AD, Alzheimer’s Disease.

A Taiwanese cohort of 17,425 PCa patients from the Longitudinal Health Insurance Database for Catastrophic Illness Patients (part of the Taiwan National Health Insurance Research Database) has been analyzed. Patients were divided by ADT treatment: 12,740 (73.1%) with ADT and 4,685 (26.9%) without ADT (9). Patients in the ADT group could have been subjected to bilateral orchiectomy or medical treatment (LHRH agonist, anti-androgens, or combination therapy). A multivariable Cox model, adjusted for age, urbanization, occupation and comorbidities was used to generate hazard ratios (HRs) of cognitive decline. ADT showed a significant association with overall risk of cognitive decline (HR = 1.51, 95% CI: 1.31 -1.74). There was no apparent correlation between duration of ADT exposure and cognitive dysfunction. Finally, when stratified based on type of ADT regimens, anti-androgen-only therapy (n= 6008) displayed a significantly higher risk of subsequent dementia (HR = 1.54, 95% CI 1.29–1.84) while GnRH agonists use and orchiectomy showed no difference in risk of cognitive dysfunction compared with patients who did not receive ADT. These results raise the concern that different ADT therapies may have disparate impacts on cognitive function.

Using the Surveillance, Epidemiology and End Results (SEER)-Medicare linked database of the National Cancer institute, three different research groups used variable models to estimate HR with 95% CI for AD or dementia among PCa patients, providing evidence that the risk of AD/dementia is associated with the use of ADT. First, Nguyen et al. included 201,797 patients in their analysis of whom 94,528 patients received LHRH agonists or antagonists. They then ascertained the long-term treatment related side effects that occurred during 19 years of follow-up, including the risk of dementia. It appeared that it was highest in those who received ADT compared with those who did not (HR 1.16, 95% CI 1.13-1.20) (13). Then, Krasnova et al. using a cohort of 100,414 men with PCa, 38% (n=37,911) of whom receiving ADT, showed that the use of ADT was associated with a 17% higher risk of all cause dementia (HR 1.17, 95% CI 1.07-1.27) and 23% higher risk of AD (HR 1.23, 95% CI 1.11-1.37) (12). Finally, evaluating the electronic medical records of 154,089 individuals, including 62,330 PCa patients (40% receiving ADT) followed for a median follow-up of 8.3 years, Jayadevappa et al. observed a statistically significant positive association between use of ADT and occurrence of AD (13.1% vs 9.4%; difference, 3.7%; 95%CI, 3.3%-3.9%; P <0.001; HR = 1.14; 95% CI, 1.10-1.18) or dementia (21.6% vs 15.8%; difference, 5.8%; 95% CI, 5.4%-6.2%; P <0.001; HR = 1.20; 95% CI, 1.17-1.24) (10).

A Korean study supported this positive association between ADT and dementia, analyzing data from the National Health Insurance Service database of the entire Korean adult prostate population (14). On a cohort of 37,549 individuals with PCa, 24,929 (66.4%) underwent ADT. With a mean follow-up of 4.1 years, a statistically significant association was found between ADT and the development of cognitive dysfunction (HR, 1.169, 95%CI 1.077-1.270).

However, two recent and large sample size cohorts did not support the correlation between the use of ADT and an increased risk of dementia. In the largest population-based cohort study to date evaluating 1.2 million fee–for-service Medicare beneficiaries age ≥ 67 years with PCa with a mean follow-up of 5.5 years, no effect of ADT on dementia risk was revealed (8). Using the United Kingdom’s Clinical Practice Research Datalink, a cohort of 30,903 men diagnosed with non-metastatic PCa was assembled and observed (11). During a mean follow-up of 4.3 years, 17,994 patients (58.2%) used ADT. This treatment was not associated with an increased risk of dementia (HR 1.02, 95% CI 0.87-1.19). Secondary analyses assessing whether the risk of dementia varied by ADT type were also negative.

In an attempt to perform a well-designed quantitative synthesis to summarize the risk of dementia and/or AD disease with ADT use, a systematic review and meta-analysis of the literature was conducted very recently by Sari Motlagh et al. (15). ADT consisted of GnRH agonists or antagonist (use of anti-androgens only or orchiectomy were excluded) and the control group included only PCa patients. By pooling the results of 14 studies, the authors found that the risk of all cause dementia and/or AD was increased in patients with PCa who receive ADT compared to those who do not receive ADT with a lower effect for AD than for dementia (HR 1.16 vs 1.21, respectively). They also examined whether ADT duration could be correlated with the new onset dementia and/or AD. They found that patients who receive ADT for 12 months or more have a statistically significantly increased risk of new onset dementia compared to those who receive a short duration. On the other hand, the risk of developing AD was not associated with ADT duration. Though the main limitation of this study is its retrospective nature, it strengthens the evidence towards a correlation between ADT and new onset of dementia.

**Figure 1:**
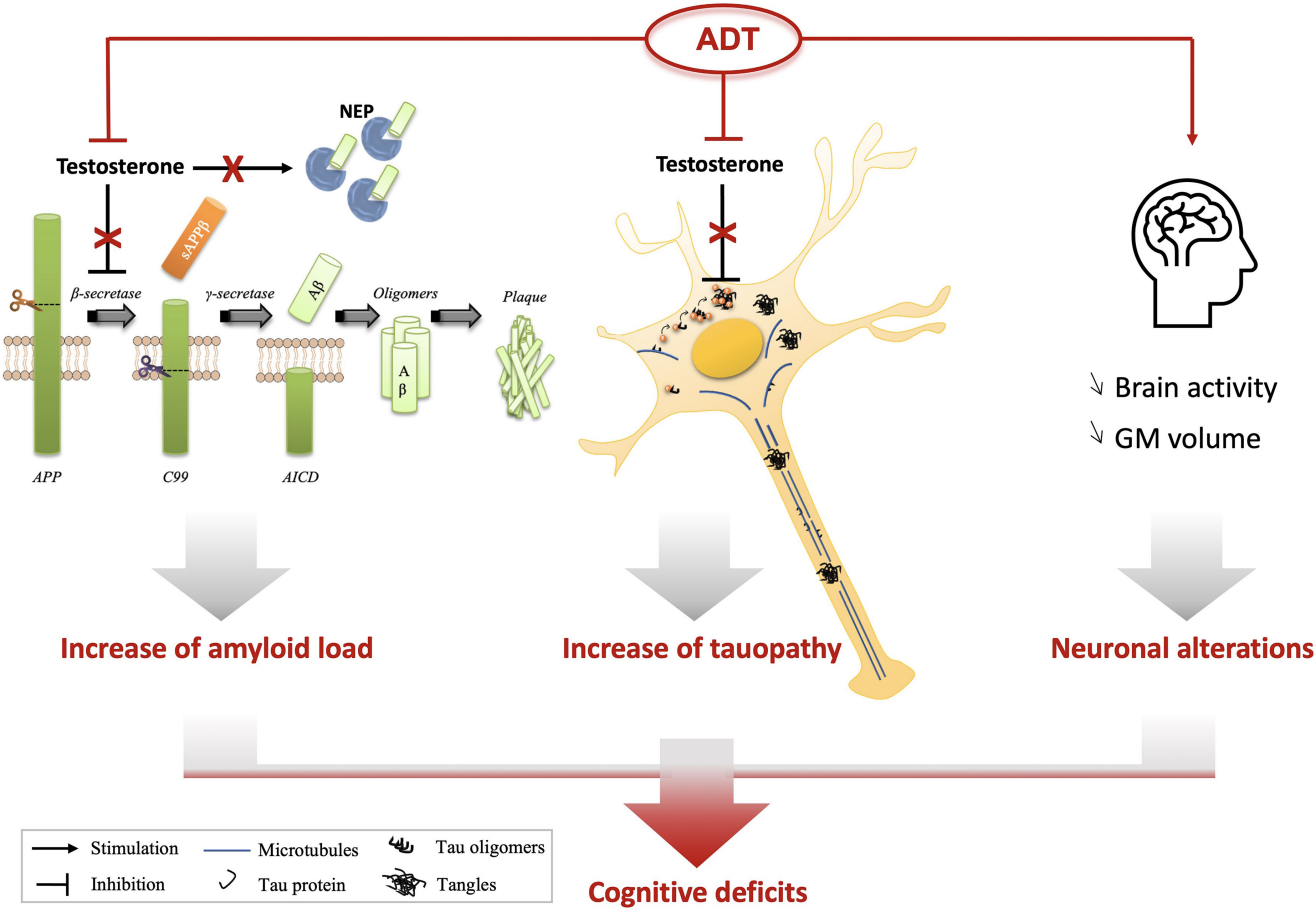
Possible connections between ADT and cognitive decline. Thanks to preclinical studies based on androgen deprivation or testosterone supplementation, it has been suggested that testosterone depletion could play a direct role on Aβ accumulation through the modulation of its production [activation of the β-secretase (BACE1)] or its degradation (reduction of neprilysin (NEP) expression and activity). Androgen deprivation also led to a decrease of hyperphosphorylation of Tau proteins (p-Tau) and their accumulation in neurons. In patients, ADT has been shown to induce a decrease of grey matter (GM) volume and a reduced brain connectivity using imaging, two characteristics also observed in AD brains. Aβ accumulation or p-Tau increase have been associated with a reduction of cognitive performances of animals, suggesting that ADT directly influences amyloid and tau levels, in addition to neuronal activity which could lead to cognitive decline of patients. APP, Amyloid Precursor Protein; AICD, APP Intracellular Domain.

These observational cohort studies, derived from existing clinical data are simpler to conduct than randomized clinical trials. However, caution should be applied when considering their results due to the inherent challenges of a big data research approach. These challenges have been well described by Nead (16) and consist in pooling potentially heterogenous data (which can result in averaging out the true effect), reliance on claims or electronic medical records (aimed to aid reimbursements and clinical care rather than research) and use of methodological choices to assess the validity of such type of studies (17). These inherent limitations could explain the contradictory results presented here. The conflicting clinical results can be balanced by biological changes observed in men undergoing ADT, as shown by brain imaging, and by the demonstration, in preclinical studies, that testosterone depletion is associated with the development of AD pathognomonic lesions. These two aspects, increasing the plausibility for an association between ADT and dementia, will be described in the following sections (**Figure 1**).

### Use of ADT and Functional and Structural Brain Imaging in PCa Patients

Functional magnetic resonance imaging (fMRI) and [^18^F]FDG PET are emerging non-invasive imaging techniques used to study neural activation changes, with some evidence provided even in PCa patients undergoing ADT (**Table 2**). Overall, none of these studies showed significant cognitive changes after ADT but changes in brain function and structure could be documented (23–27).

**Table 2:** Imaging studies investigating changes in neuronal activation in response to androgen deprivation therapy in prostate cancer patients.

Author (reference no.)	Number of participants	Neurocognitive Tests	Type of imaging	ADT type	Main findings
**Cherrier et al.** **2010** (18)	5 PCa patients treated with ADT *Vs* 7 control healthy patients	Spatial reasoning and spatial memory task: 1/Encoding task (ENCODE) 2/Recognition task (RECOG) 3/Mental Rotation Matching Task (MATCH)	(BOLD)-fMRI: • prior to the start of ADT (time1) • after 9 months of ADT (time2)	9 mo of Leuprolide 7.5 mg	Decrease right parietal activation with ADT (at time 2 for RECOG and MATCH but not ENCODE, with no difference in scanner behavioral performance)
**Chao et al.** **2012** (19)	15 PCa patients treated with ADT *Vs* 15 control untreated PCa patients	1/N-back (working memory) task (outside MRI scanner) 2/Stop signal (cognitive control) task	(BOLD)-fMRI: • prior to the start of ADT (time1) • after 6 months of ADT (time2)	6 mo of Goserelin 10.8 mg	Decrease in medial prefrontal cortex, right insula and right middle/inferior frontal cortices activations with ADT
**Chao et al.** **2013** (20)	12 PCa patients treated with ADT *Vs* 12 control untreated PCa patients	1/N-back (working memory) task (outside MRI scanner)	Voxel-based Morphometry (VBM): • prior to the start of ADT (time1) • after 6 months of ADT (time2)	6 mo of Goserelin 10.8 mg	Decrease of GMV in fronto-polar cortex, dorsolateral prefrontal cortex and primary motor cortex with ADT
**Cherrier et al., 2018** (21)	9 PCa patients treated with ADT	Cognitive and mood measure at baseline and 9 mo	18F-FDG-PET: • prior to the start of ADT (time1) • after 9 months of ADT (time2)	Flutamide 250 mg TID and leuprolide acetate 7.5 mg monthly for a total of 9 mo	Decrease in brain metabolism in posterior cingulate, cerebellum and thalamus with ADT
**Plata-Bello et al., 2019** (22)	50 PCa patients treated with ADT ≥ 6 mo *Vs* 15 control untreated PCa patients	Neuropsychological assessment	MRI with T1 and FLAIR sequences	NA	Negative relationship between the ADT duration and the GMV

MRI, magnetic resonance imaging; ADT, androgen deprivation therapy; GVM, grey matter volume.

In a study by Cherrier et al. (25), 5 PCa patients with biochemical relapse following primary therapy without evidence of metastases were treated with 9 months of ADT (leuprolide). fMRI of the brain during spatial reasoning and memory tasks was performed before the treatment and after 9 months of ADT. Seven healthy patients, matched for age and education, underwent fMRI at the same time intervals. Chemically-castrated patients showed reduced task-related fMRI activation compared to control subjects, not associated with cognitive changes.

Chao et al. observed similar results in a prospective observational cohort analysis of men with non-metastatic PCa at a Veterans Affairs medical center (24). Fifteen patients receiving 6 months of Goserelin (as adjuvant treatment or because of biochemical recurrence) were assessed by fMRI and for cognitive functions at baseline (prior ADT) and after 6 months of ADT. The control group consisted of 15 non-metastatic PCa patients who had never been treated with ADT, matched for age and level of education. While no differences were observed between groups for cognitive function, prefrontal cortical activations were impaired on fMRI in patients receiving ADT. In a subsequent analysis implementing structural MRI at baseline and 6 months (23), a significant decrease in the gray matter volume in the fronto-polar, the dorsolateral prefrontal, and the primary motor cortex regions was observed in PCa patients undergoing ADT compared to controls.

Using [^18^F]FDG PET imaging in 9 PCa patients treated with ADT for a biochemical recurrence, Cherrier et al. observed a decreased regional cerebral glucose metabolism in the cerebellum, the posterior cingulate and the medial thalamus bilaterally after 9 months of androgen ablation compared to the pretreatment baseline findings (26).

More recently, Plata-Bello et al. conducted a transversal analysis on 65 PCa patients, 50 of whom were treated with ≥ 6 months of ADT (27). Every patient underwent a cerebral MRI with measures of the grey matter volume (GMV) and white matter lesion (WML) load. Compared to the 15 controls not receiving ADT, the GMV and WML burden were similar in the two groups, but a negative relationship between the ADT duration and the GMV was identified. The authors concluded that PCa patients exposed to ADT may present an acceleration of age-related brain changes, including WML development and GMV loss.

Findings from these studies must be interpreted with caution due to the small size of the PCa patients’ cohorts. Results should be replicated with more subjects. However, all these studies consistently show a change in brain activity as measured by PET or MRI imaging in men with PCa undergoing ADT. The overlap of brain regions with changes in neuronal activation in ADT and early AD suggests possible common mechanisms. This idea is reinforced by experiments in small animals. Though the pathogenesis of AD is complex and multifactorial, loss of testosterone has been shown *in vivo* in animal models of AD to be linked with AD pathogenesis and this will be the subject of our next section.

### Testosterone Deprivation and Alzheimer’s Disease Pathogenesis in Small Animals

AD is characterized from a pathological point of view by two pathognomonic lesions: 1/extracellular plaques of insoluble β-amyloid peptides (Aβ) and 2/neurofibrillary tangles composed of hyperphosphorylated tau protein (p-tau) in neural cytoplasm. Using preclinical experiments in rodents, testosterone has been shown to modulate Aβ and potentially hyperphosphorylation of tau in the serum and the central nervous system.

#### The Role of Testosterone Depletion on Aβ Accumulation

Ramsden et al., comparing gonadectomized (GDX) and sham GDX male rats, were the first to show that depletion of endogenous sex steroid hormones *via* GDX caused a 25% increase in soluble brain levels of Aβ40 and Aβ42. They also revealed that injections of 5α-dihydrotestosterone (DHT) to GDX rats not only reversed the GDX-induced increase in Aβ but significantly lowered brain levels of both Aβ species compared to sham GDX animals. In contrast to the increase in soluble brain levels of Aβ, no effect of GDX on plasma Aβ was observed (28).

Rosario et al. used a triple transgenic mouse model of AD (3xTg-AD) (29) and repeated the experience, comparing Aβ accumulation in subiculum, CA1 region of the hippocampus and amygdala between GDX and sham GDX mice and evaluating the adjunction of DHT to GDX mice for Aβ brain accumulation. They also compared behavioral performances with a hippocampal-dependent task of working memory. They showed an increase of Aβ load in all brain regions for GDX mice compared to non GDX mice in parallel with a significantly impaired task of working memory. DHT treatment of GDX mice attenuated both Aβ accumulation and memory deficits (30).

The effect of testosterone on the level of Aβ peptides was also investigated by Wahjoepramono et al. in guinea pigs (31). In accordance with the previous studies, castrated guinea pigs (GPX) exhibited lower cerebrospinal fluid (CSF) Aβ40 level that non castrated animals. But, unlike Ramsden et al., these authors showed also a decrease in plasma Aβ40 level for GPX.

If the link between testosterone depletion in plasma and brain Aβ accumulation has been pointed out, the underlying molecular mechanisms are still unclear.

The Aβ-catabolizing enzyme neprilysin (NEP) could be involved in this process. NEP is a regulator of Aβ in brain and is involved in Aβ clearance. Depletion of NEP results in neural accumulation of Aβ. In an animal study, Yao et al. (32) demonstrated that testosterone function as endogenous regulator of Aβ in brain *via* androgen receptor (AR)-dependent regulation of NEP. Indeed, DHT induced an increase in NEP expression and a decrease in levels of Aβ in AR expressing cells. The DHT-induced decrease of Aβ was blocked by inhibition of NEP.

In addition to the upregulation of NEP, endogenous testosterone may protect against AD in males *via* downregulation of β-site APP cleaving enzyme 1 (BACE1) activities at transcriptional level to reduce Aβ production (33). Indeed, Aβ is generated from cleavages from the amyloid precursor protein (APP) (34) and BACE1 is known to cleave APP at the N-terminal end of the Aβ sequence (35). When comparing the transgenic APP23 mouse model of AD to mice with increased endogenous testosterone (APP23/Ar^+/-^), McAllister et al. found that APP23/Ar^+/-^ mice presented significant reduction in brain plaque formation, increased NEP activity and reduced BACE1 enzyme activity (33).

The role of luteinizing hormone (LH) in regulation of Aβ has also been investigated by Rosario et al. (36). Indeed, like low testosterone, the age-related increase in LH has been linked to AD in men (37–40). It is unclear whether changes in testosterone or LH levels primarily underlined the relationship with AD. In order to solve this issue, the authors compared levels of Aβ in male 3xTg-AD mice under varying conditions. They first treated gonadally intact mice with leuprolide, thus lowering both testosterone and LH. They observed an increase in Aβ accumulation. They also observed an increase in Aβ accumulation in GDX mice who present low testosterone but high LH. Treatment of GDX mice with testosterone significantly reduced Aβ levels. But treatment of GDX mice with leuprolide did not significantly decrease Aβ levels. These findings suggest that the Aβ lowering action of testosterone is mediated by the androgenic pathways rather than *via* regulation of LH and the hypothalamic-pituitary-gonadal axis.

Finally, testosterone injections improved cognitive performance in male rats induced by intrahippocampal injections of Aβ42 oligomers and markedly decreased the hippocampal protein expression level of Aβ (41, 42). Flutamide, an anti-androgen, inhibited all of the testosterone-mediated effects. These data underline the fact that the influence of testosterone on cognitive performance is mediated *via* an AR pathway to remove Aβ.

#### The Role of Testosterone Depletion on Tau Hyperphosphorylation

The role of testosterone depletion on tau hyperphosphorylation has been less studied than on Aβ accumulation. Rosario et al. found a statistically non-significant increase in the level of tau hyperphosphorylation in GDX male 3xTg-AD mice compared to sham GDX animals (43), suggesting that testosterone depletion does not regulate tau pathology. On the contrary, Papasozomenos et al. showed that for GDX male rats, there was hyperphosphorylation of tau 3 hours after heat shock, which induces rapid dephosphorylation of tau. The hyperphosphorylation was not observed in GDX male rats previously treated with testosterone propionate (44). Finally, the existence of an interaction between testosterone and glucocorticoids in the regulation of tau hyperphosphorylation and accumulation in male rats brain has been recently suggested by Monteiro-Fernandes et al. (45). While prolonged exposure to glucocorticoids trigger tau hyperphosphorylation and its accumulation into axonal and somatodendritic compartments, these glucocorticoids actions seems to be countered by testosterone.

## Alzheimer’s Disease Biomarkers

**Figure 2:**
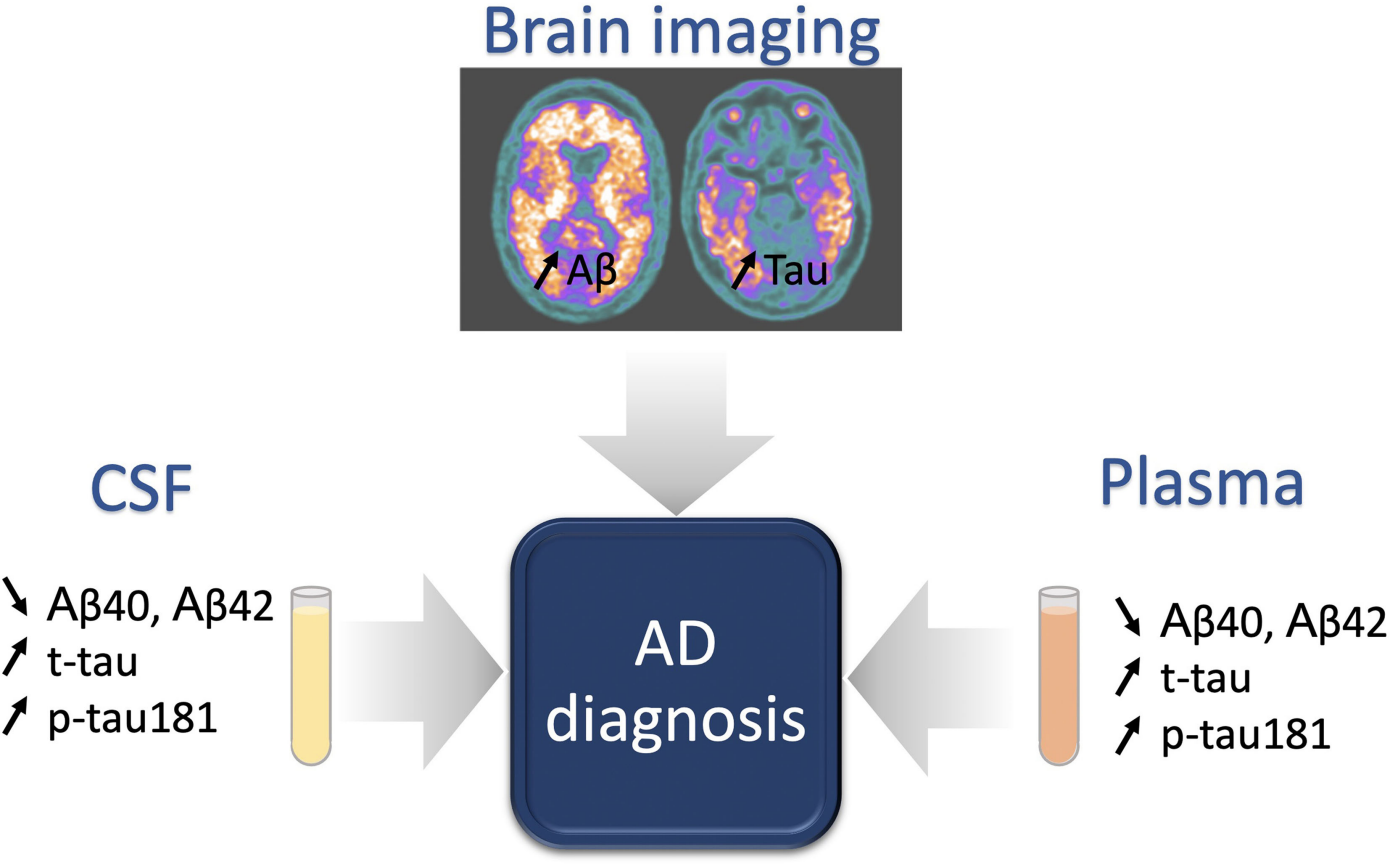
Classical biomarkers for AD diagnosis. Brain imaging and fluid biomarkers are under intense development for AD diagnosis. An increase of amyloid deposits and tauopathy are observed in the brain of AD patients with a well-defined pattern, evolving during pathology progression. Tauopathy biomarkers, including classically the total tau (t-tau) and phosphorylated Tau on Threonine 181 (p-tau181), follow the same profile of expression in fluids (cerebrospinal fluid (CSF) and plasma), with an increase of concentration during the pathology. At the opposite, low levels of amyloid peptides (Aβ40 and Aβ42) in the CSF or in the plasma reflect high amyloid deposition in the brain.

Imaging and fluid biomarkers have emerged in the last two decades as useful diagnostic and prognostic tools for AD evaluation, as they allow *in vivo* assessment of AD-specific pathophysiological hallmarks (**Figure 2**), namely amyloid and tau, as well as non-specific but related phenomena: neurodegeneration, synaptic loss, neuroinflammation. While an extensive description is beyond the scope of this review, we’ll briefly summarize here the clinically available biomarkers to measure amyloid and tau pathology in patients.

Molecular imaging by positron emission tomography (PET) using dedicated tracers allows visualizing the brain deposits of amyloid plaques and neurofibrillary tangles of hyperphosphorylated tau. Multiples studies with neuropathology as gold standard have shown that PET imaging has good to excellent sensitivity and specificity to detect the pathological processes (46, 47).

In the cerebrospinal (CSF) fluid it is possible to measure total tau (t-tau) or different isoforms of phosphorylated tau, namely p-tau181, and amyloid markers, in particular Aβ42 or the relative load of Aβ42 and Aβ40 (48). Indeed, a marked decrease in CSF Aβ42 and a marked increase in CSF t-tau and p-tau are observed in symptomatic AD patients (49–51). Even if significant advances have been made in standardization of CSF sampling and protocols for biomarker measurement, no consensus on cut-off values has been defined yet (52).

A growing interest for blood biomarkers use in AD appears these last years, mainly due to the fact that plasma markers are highly accessible, non-invasive and less expensive than PET or CSF examinations, and thus represent the ideal tool for screening or for longitudinal observations in large populations (53). As with CSF biomarkers, Aβ42 and Aβ40 are the most studied blood biomarkers for the diagnosis of AD. This field has dramatically progressed over the last few years with the development of ultra-sensitive assays able to reliably measure the low levels of circulating biomarkers. A first study showed in 2017, that plasma levels of Aβ42 and Aβ40 were reduced in AD dementia compared with subjects with mild cognitive impairment (MCI) or a subjective cognitive decline (54, 55). The combination of decreased plasma Aβ42/(APP)_669-711_ and Aβ42/Aβ40 ratios proved to be predictive of brain Aβ burden assessed by [^11^C]PIB (Pittsburgh compound B)-PET imaging (56). Plasma Aβ42/Aβ40 had a high correspondence with amyloid PET status and CSF p-tau181/Aβ42 levels (57). Moreover, individuals with a negative amyloid PET scan at baseline and a positive (= low) plasma Aβ42/Aβ40 had a 15-fold greater risk of conversion to amyloid PET positive compared to individuals with a negative plasma Aβ42/Aβ40 (57), showing that not only plasma Aβ42/Aβ40 could be used as a diagnostic biomarker but also as a predictive biomarker of AD. The most convincing evidence concerns the development of sensitive assays to measure p-tau isoforms in plasma. In 2017, an exploratory study showed that plasma levels of p-tau181 were significantly higher in the AD patients than those in the controls (58). The predictive value of plasma p-Tau181 for amyloid and tau brain levels was demonstrated recently (56, 59). Moreover, plasma p-tau181 has also been associated with the disease progression, as plasma p-tau181 level was increased in preclinical AD and further increased at the MCI and dementia stage (59). Plasma p-tau181 also allows to discriminate AD from other neurodegenerative disorders, with an accuracy similar to that of tau PET and CSF p-tau181 (59), and identifies AD across the clinical continuum with gradual increase: from the lowest concentrations in Aβ negative young adults and cognitively unimpaired older adults, through higher concentrations in the Aβ positive cognitively unimpaired older adults and MCI groups, to the highest concentration in the Aβ positive MCI and AD groups (60). More recent evidence shows the excellent performance of also p-tau 217 and p-tau 231 (61, 62).

Overall, blood biomarkers appear as good candidates to easily detect and even predict the progression of AD but further studies are necessary to validate their clinical use. The level of evidence for the diagnostic use of these different biomarkers has been recently revised within a systematic framework, in order to accelerate their clinical implementation (63, 64). Amyloid PET, tau PET with [^18^F]flortaucipir, the most commonly used first generation tracer, and, CSF biomarkers have shown analytical validity, i.e. an adequate level of accuracy to detect the pathology of interest, and initial evidence for clinical validity, i.e. their ability to favorably impact the diagnostic process in the prodromal stage (19, 20, 52). The level of validity for plasma markers is still lower, but this literature is rapidly evolving and the accessibility of the method is greatly facilitating its implementation in large scale studies (18).

More specifically related to ADT, no PET imaging or CSF studies measuring AD biomarkers have been reported. Only two studies evaluated the effect of ADT on plasma biomarkers. Gandy et al. studied the effect of ADT in 6 men on plasma levels of Aβ40. They found that after chemical castration, an increase of plasma Aβ40 concentration was observed for all 6 patients (21). Results were confirmed in a second study with 37 patients showing an increase of Aβ40 levels in plasma, in addition to higher anxiety and depression scores. Interestingly, the discontinuation of the androgen-deprivation was associated with an improvement of general cognitive performances (Cambridge Cognitive Examination for the Elderly score) (22). Larger studies and the measurement of other biomarkers are consequently necessary to interpret these results.

## Discussion: Clinical Applications And Future Perspectives

### Clinical Applications

#### Patient Counselling

The international Society of Geriatric Oncology guidelines of 2019 recommend that clinicians should discuss the risk of cognitive dysfunction with elderly PCa patients who are considered for ADT (65). This recommendation was mainly based on a meta-analysis of the retrospective cohort studies by Sun et al. published in 2018. In this study, the authors found a higher, but not statistically different, risk of cognitive impairment after ADT (HR 1.28, 95% CI 0.93-1.76) (66). However, this meta-analysis included few studies which assessed dementia and/or ADT as an outcome with an estimated risk effect (quantitative synthesis) and the analyzed cohort studies had different outcome definitions. The more recent meta-analysis from Sari Motlagh et al. that does not suffer these limitations, established a stronger link between ADT use and the development of AD/dementia (15).

These results could contribute to the creation of new guidelines recommending not only to discuss the risk of cognitive dysfunction with PCa patients considered for ADT but also to assess patient’s cognitive status at baseline, thorough, and after completion of ADT. However, unless the association between ADT and AD/dementia is confirmed in prospective studies, this potential risk should be communicated and discussed with the patient with caution, especially for men who will clearly derive a survival benefit from the hormonal treatment.

#### Assessment of Patient’s Cognitive Status

Before prescribing ADT, the physician has to carefully report the inherent patient’s risk factors for AD development. Genetics has a key role in the development of AD, but acquired factors have also been identified, such as cerebrovascular diseases, diabetes, hypertension, obesity, and dyslipidemia (67). The cognitive status should also be systematically assessed at baseline in all patients planned for ADT and during the course of ADT. A large number of screening instruments for AD are available (68), as the Montreal Cognitive Assessment (MoCA) which seems to be a promising screening test due to its high sensitivity (84%) and specificity (74%) values (69).

The assessment of specific Aβ and p-tau biomarkers before and throughout ADT could also be proposed. This would allow confirming the alleged role of ADT on the new onset of AD by assessing the cognitive impact of ADT prospectively. This is of crucial importance since the trend is to intensify ADT by adding second generation anti-androgens for high-risk prostate cancer patients as evaluated by ongoing studies (ATLAS, NCT02531516 and ENZARAD, NCT02446444).

In a second time, if the correlation between ADT use and neurodegenerative disorders is confirmed, then biomarkers could help the physician to estimate an individualized balance benefit/risk taking into account both the risk of developing AD and the risk of death/disability from PCa disease.

#### ADT and Radiotherapy: Patient Selection and Duration

Careful selection of PCa candidates for concomitant ADT is essential to minimize the risk of cognitive disorders associated with androgen ablation. Moreover, efforts to shorten the duration of ADT especially for long-term ADT protocols combined with curative radiotherapy may help to limit the associated toxicities of this treatment. By reducing from 36 to 18 months the duration of ADT, a randomized phase III trial demonstrated that outcome of high-risk PCa patients was not impaired while quality of life was significantly improved with the shortest schedule (70). How further reduction in ADT duration can be safely proposed in frail and dementia-prone PCa patients remains an open question, considering that in meta-analysis by Sari Motlagh et al. the risk of dementia was higher in patients who received ADT for more than 12 months (15). Moreover, the association between new LH-RH antagonists and modern androgen receptor inhibitors and the risk of long-term neurodegenerative disorders remains presently unclear and certainly deserves further investigations.

### Future Perspectives

ADT is widely used in PCa treatment (71, 72). Studies in animals and human point to an increased risk of altered cognitive function linked to the use of ADT through a number of different mechanisms, both direct and indirect, as summarized in **Figure 1**. However, several limitations weaken the quality of the evidence available. First, there are inherent weaknesses in the application of retrospective analyses investigating the association between ADT and the risk of dementia in large electronic patient’s databases, which have been described above. Second, the only brain imaging studies addressing neuronal changes in PCa patients undergoing ADT have not included AD-specific imaging biomarkers and focused on small numbers of patients. As a consequence, better tools are needed to assess the cognitive impact of ADT prospectively in order to optimize PCa patients care. Further preclinical and clinical investigations with integration of metabolic AD specific imaging and blood biomarkers are expected to be very useful in this setting. Finally, specific studies should target the pathophysiological changes associated with different androgen-deprivation strategies, including the new LH-RH antagonists and modern androgen receptor inhibitors.

## Conclusions

Neurocognitive impairment is emerging as a potential additional long-term side effect of androgen suppression in PCa patients. Due to the great potential impact on quality of life, clear evaluation of treatment indications and appropriate counselling and follow-up of cognitive status is recommended for PCa patients undergoing ADT, especially with long-term or long-life regimens. Further preclinical and clinical investigations integrating metabolic imaging and modern biomarkers are eagerly warranted to understand underlying mechanisms and to better assess the impact of ADT on long-term neurocognitive function.

## Author Contributions

TZ and VG contributed to conception and design of the study. VA wrote the first draft of the manuscript. KC created the figures. All authors contributed to manuscript revision, read, and approved the submitted version.

## Funding

This work was supported by the Velux foundation (grant number 1123). Granting bodies were not involved in the drafting of the manuscript. TZ was funded by the Swiss National Science Foundation (project 320030_182366). VG was funded by the Swiss National Science Foundation (projects 320030_169876 and 320030_185028).

## Conflict of Interest

The authors declare that the research was conducted in the absence of any commercial or financial relationships that could be construed as a potential conflict of interest.

## Publisher’s Note

All claims expressed in this article are solely those of the authors and do not necessarily represent those of their affiliated organizations, or those of the publisher, the editors and the reviewers. Any product that may be evaluated in this article, or claim that may be made by its manufacturer, is not guaranteed or endorsed by the publisher.
